# Molecular Epidemiology of Glanders, Pakistan

**DOI:** 10.3201/eid1512.090738

**Published:** 2009-12

**Authors:** Heidie Hornstra, Talima Pearson, Shalamar Georgia, Andrew Liguori, Julia Dale, Erin Price, Matthew O’Neill, David DeShazer, Ghulam Muhammad, Muhammad Saqib, Abeera Naureen, Paul Keim

**Affiliations:** Northern Arizona University, Flagstaff, Arizona, USA (H. Hornstra, T. Pearson, S. Georgia, A. Liguori, J. Dale, M. O’Neill, P. Keim); The Translational Genomics Research Institute, Phoenix, Arizona (E. Price, P. Keim); US Army Medical Research Institute of Infectious Diseases, Fort Detrick, Maryland, USA (D. DeShazer); University of Agriculture, Faisalabad, Pakistan (G. Muhammad, M. Saqib); Veterinary Research Center, Sultanate of Oman (M. Saqib); University of Veterinary and Animal Sciences, Lahore, Pakistan (A. Naureen)

**Keywords:** Burkholderia mallei, glanders, equidae, VNTR, MLVA, bacteria, molecular epidemiology, Pakistan

## Abstract

We collected epidemiologic and molecular data from *Burkholderia mallei* isolates from equines in Punjab, Pakistan from 1999 through 2007. We show that recent outbreaks are genetically distinct from available whole genome sequences and that these genotypes are persistent and ubiquitous in Punjab, probably due to human-mediated movement of equines.

Glanders is an equine disease that was recognized by Hippocrates and Aristotle (*1*). It is caused by the bacterium *Burkholderia mallei*, an obligate pathogen of horses, donkeys, and mules (Equidae), with occasional infections in felines, canines, and humans (*2**,**3*). Strict regulations of equines have reduced the range of this once globally distributed disease to a few endemic foci in South and Central America, the Middle East, and parts of Africa and Asia (*2**,**4*). This emerging disease has only recently regained attention following the listing of *B. mallei* as a Category B agent by the US Centers for Disease Control and Prevention (*2*). Although outbreaks are common in regions of disease endemicity, much of what is known about the ecology and natural population dynamics of *B. mallei* relies on indirect evidence and expert opinion, with little to no knowledge concerning its genetic diversity (*2**,**3*). We genetically characterized 15 samples of *B. mallei* from recent outbreaks in Pakistan to provide additional knowledge of how this disease of antiquity is transmitted throughout endemic regions today.

## The Study

We obtained clinical samples and background information from 15 glanderous equids in Punjab, Pakistan from 1999 through 2007 (Table; Appendix Table). Research on equine subjects was approved by the Synopsis Scrutiny Committee and Animal Ethics Committee, Faculty of Veterinary Science, University of Agriculture, Faisalabad, Pakistan. Samples were plated on brain-heart infusion (BHI) agar with 4% glycerol and incubated for 24–30 hours at 37°C. Individual colonies were inoculated into BHI broth containing 4% glycerol and were incubated with shaking for 36 h at 37°C. An aliquot of broth (1.5 mL) was centrifuged at 13,000 rpm for 15 min. Genomic DNA was extracted from the resulting pellets using standard digestion buffer and phenol-chloroform extraction protocols (*6*).

**Table:** Spatial, temporal, and phylogenetic relationships among *Burkholderia mallei* infections in equids, Punjab Province, Pakistan*

Epidemiologic group and subgroup designation (isolate names)†	Description	Clade(s)‡	Total VNTR differences between subgroups
Group 1 Group 1a (PRL2) Group 1b (PRL11, PRL13)	Strains were collected from an outbreak among Faisalabad Mounted Police Horses (n = 18) in June 1999. Biochemical test results (based on Analytical Profile Index 20E strips; BioMériux, Marcy l’Etoile, France) differed nonsubstantially (data not shown). Therefore, only 3 isolates were evaluated by using VNTR. Strains PRL11 and PRL13 were isolated from horses that were kept at 2 stables ≈8 km away from each other, but the 2 stables had a history of mixing.	A	3/15 loci
Group 2 Group 2a (PRL42) Group 2b (PRL45)	Samples came from 2 sporadic cases of glanders in draught mules from the Faisalabad district in 2007. Reports indicate that these animals drank from communal water troughs available in different zones of Faisalabad.	C	1/15 loci
Group 3 Group 3a (PRL1) Group 3b (PRL41) Group 3c (PRL7)	Samples came from 3 sporadic cases of glanders in draught equids from the Faisalabad district during different years (2002, 2006, and 2000, respective to subgroup listing). Reports indicate that these animals drank from communal water troughs available in different zones of Faisalabad.	B§ B, no clade¶	2/15 loci§ 14/15 loci¶
Group 4 Group 4a (PRL33) Group 4b (PRL34)	Samples were obtained from 2 donkeys that worked and were housed together in a brick factory in the district of Faisalabad. Samples were collected 3 weeks apart in 2007, and the strains were passaged 3× in guinea pigs before DNA was extracted for VNTR evaluation.	A	3/15 loci
Group 5 Group 5a (PRL19) Group 5b (PRL20)	In September 2005, an outbreak of glanders occurred at the Lahore Polo Club. Two isolates were obtained from separate horses in this group, and each isolate had a different biochemical profile (data not shown).	A, B	8/15 loci
Group 6 Group 6a (PRL3, PRL4) Group 6b (PRL44)	From November 2004 to March 2005, two horses from a farm in Sargodha (PRL3, PRL4) participated in matches at the Lahore Polo Club. Horses were returned to their farm in late spring 2005. In the fall of 2005, there was a glanders outbreak at the Lahore Polo Club (see Group 5 above). In December 2005, the 2 horses on the Sargodha farm tested positive for glanders after being housed together during the winter. A mule (PRL44) that was also present at the Sargodha farm tested negative for glanders at this time. Approximately 2 years later, the same mule tested positive for glanders after reports of 6 months’ standing nasal discharge. Records indicate the mule was brought to the farm at a young age from the city of Multan and never left the farm before onset of symptoms.	A	4/15 loci

*VNTR, variable number tandem repeats. †Samples from equines with similar histories were assigned to the same epidemiologic group (e.g., Group 1, Group 2). Subgroups were defined based on VNTR data; samples with identical VNTR genotypes were assigned to the same subgroup (e.g., PRL11 and PRL13 in Group 1b). ‡See Figure 2. §Data are comparing subgroups 3a and 3b to each other (PRL1 and PRL41) and excluding subgroup 3c (PRL7). ¶Data are comparing subgroups 3a and 3b combined (PRL1 with PRL41) to subgroup 3c (PRL7).

For genotyping, we screened 23 loci (Appendix Table) from a previously established 32-marker multiple locus variable number of tandem repeats (VNTR) analysis system designed for *B. pseudomallei* and *B. mallei* (*7*). *In silico* genotyping of the same loci was also performed for 10 whole genome sequences (WGS) of *B. mallei* (*8*; Appendix Table). VNTR markers have higher mutation rates than other genetic markers which make them inappropriate for determining deep levels of evolutionary relatedness, however VNTRS are appropriate for 1) discrimination between closely related isolates, 2) determination of the degree of relatedness among isolates, and 3) discernment of population structure on a spatial scale (*7**,**9**,**10*). This utility is especially important for *B. mallei* because it is a recently emerged clone of *B. pseudomallei* and has been shown to be genetically monomorphic with typing methods such as multilocus sequence typing (*11*). To compare the genetic diversity of our Punjab isolates to that of sequenced strains, we performed a phylogenetic analysis on the 23 loci using the neighbor-joining algorithm in PAUP* 4.0b (*9*). To determine the genetic relationships among the Punjab population itself, we performed the same analysis using only the Punjab isolates and polymorphic loci (n = 15 loci).

**Figure 1:**
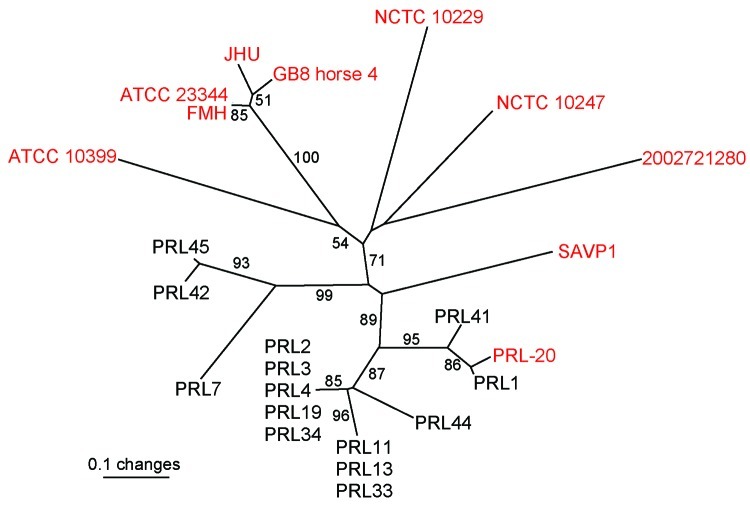
Unrooted neighbor-joining tree based on 23 variable number tandem repeat loci demonstrating that the Punjab isolates (black text and PRL-20) are genetically distinct from and less diverse than available whole genome sequences (WGS, red text) (*8*). Statistical supports for branches based on 1,000 bootstrap iterations are shown. Sample PRL-20 is shown in red text because it is also available as a whole genome sequence; therefore, it was used in all 3 situations where an average pairwise distance (APD) was calculated. Among 10 WGS, the average pairwise distance was 0.607; between 10 WGS and Punjab isolates, average pairwise distance was 0.627; and among 15 Punjab isolates, average pairwise distance was 0.312. These results indicate that the Punjab isolates are more closely related to each other than to the sequenced strains because the APD among Punjab isolates is 2× lower than the APD calculated in the other 2 situations.

**Figure 2:**
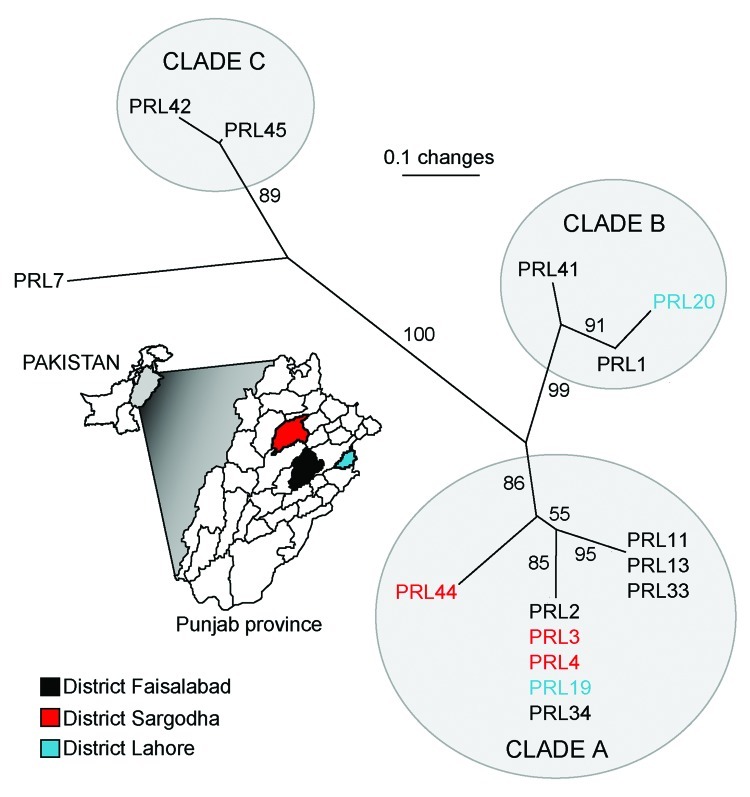
Unrooted neighbor-joining tree showing phylogenetic relationships among 15 samples of *Burkholderia mallei* from the Punjab Province, Pakistan. Statistical support for each branch derived from 1,000 bootstrap iterations are shown. Sample names are color-coded to match their district of origin in reference to the inset map of the Punjab Province. Approximate linear distances between districts are Faisalabad to Lahore ≈120 km, Faisalabad to Sargodha ≈84 km, Lahore to Sargodha ≈168 km.

Combined analysis of the Punjab isolates and WGS showed that the Punjab isolates are phylogenetically distinct from WGS (Figure 1). This finding was also demonstrated in the values for average pairwise distance (APD), where the APD among Punjab isolates is 2× lower than the APD calculated for either the entire phylogeny or the WGS alone (Figure 1). Therefore, the Punjab isolates represent only a small amount of the genetic diversity demonstrated in this pathogen. Phylogenetic analysis of the Punjab isolates alone placed 14 of the 15 samples into 3 distinct clades with 1 sample standing alone (Figure 2). Most samples (9/15) belong to clade A, whose isolates are both temporally and geographically diverse, suggesting that this lineage is ecologically established in Punjab.

Because of the limited sample size, many of the patterns observed from these data may result from sampling bias. However, even a limited amount of genotypic data can be useful in formulating hypotheses regarding the dispersal of *B. mallei*. For example, the presence of samples from Faisalabad in each clade suggests that this district may be a center of diversity in the province (Figure 2) but this does not indicate a lesser degree of diversity in other districts where fewer samples were collected.

The diversity seen in the district of Faisalabad may result from either 1) the industrial nature of Faisalabad or 2) from high endemism of *B. mallei* in the region. Currently, the district has ≈10,000 horses and mules and >44,000 donkeys, plus other transient equines (*12*). Many equines move through and work in the city, potentially introducing strains from surrounding regions. Because horses and mules can be positive but asymptomatic for glanders (*13*), many hosts are available to maintain strains throughout the region. Communal stables and water troughs are common throughout the district and *B. mallei* has been isolated from these water troughs (A. Naureen, unpub. data). Furthermore, *B. mallei* can remain viable in contaminated stables for up to 6 weeks (*14*) and in sterile tap water for up to 4 weeks (*15*), which provides an environment for establishment and retention of *B. mallei* populations in Faisalabad.

Combining phylogenetic with epidemiologic data reveals how *B. mallei* disseminates throughout a region. For example, epidemiologic data suggests that 2 horses from a farm in the district of Sargodha (PRL3 and PRL4) contracted glanders while at a polo club in the Lahore district. This is supported by VNTR data, as these 2 isolates clustered phylogenetically with one of the samples obtained from an outbreak that occurred at the same polo club 3 months prior (groups 5 and 6, Table). Furthermore, at the time of the PRL3 and PRL4 infections, a co-resident mule with no previous travel history (PRL44) was negative for glanders, making it unlikely that these horses acquired glanders from their farm. This mule was positive for glanders ≈1.5 years later, and the isolate obtained from its infection clustered phylogenetically with the samples from the polo club and Sargodha horses. Therefore, we hypothesize that the infected horses either directly transferred the disease to the mule or they contaminated a source on the farm which subsequently led to the mules infection. Environmental sampling would be required to identify the original infection source for the horses and subsequent transmission route to the mule. Nevertheless, this case shows a strain that was transferred a distance of 168 km, demonstrating that human-mediated movement of equines can influence the distribution of *B. mallei* genotypes. This case also suggests that a strain can persist for ≈1.5 years.

Other cases in the province demonstrate that infections either stem from similar strains or are caused by multiple strains. For example, samples that were placed in the same epidemiologic group cluster together phylogenetically (groups 1, 2, and 4; Table), indicating communal infections similar to the cases described above. In contrast, epidemiologic group 5 (PRL19 and PRL20) was separated into 2 distinct clades (Figure 2), indicating that this outbreak was caused by multiple strains. Therefore, it should not be assumed that an outbreak of glanders is always caused by a single strain.

## Conclusions

Our study suggests that numerous lineages of *Burkholderia mallei* are present in Punjab, Pakistan, and that these lineages persist across geographic space and time. Despite this, these isolates appear to be genetically distinct from other studied strains. The economics and use of equines likely contribute to the persistence of glanders in this region because modern methods for control of this disease (monitoring and euthanasia) are not viable options. Therefore, other solutions to curbing the spread of glanders need to be identified. We suggest that a focus on finding methods to improve the sanitary conditions of communal water troughs and stables may lead to a practical solution for disease reduction and containment. Finally, our study demonstrates the utility of VNTRs paired with extensive epidemiologic data for analyzing the distribution of *B. mallei* genotypes throughout endemic regions.

## Supplementary Material

Appendix Table Epidemiologic and 23-locus VNTR data for 15 isolates of Burkholderia mallei from the Punjab province, Pakistan, and 9 Burkholderia mallei whole genome sequences*
