# Supplementary material for: Molecular Epidemiology of Glanders, Pakistan
Source: Emerg Infect Dis. 2009 Dec;15(12):2036–9. doi: 10.3201/eid1512.090738 (PMC3044535; doi:10.3201/eid1512.090738)
Supplement: Appendix Table — Epidemiologic and 23-locus VNTR data for 15 isolates of Burkholderia mallei from the Punjab province, Pakistan, and 9 Burkholderia mallei whole genome sequences* [file 09-0738_appT-s1.pdf]

Publisher: CDC; Journal: Emerging Infectious Diseases
Article Type: Dispatch; Volume: 15; Issue: 12; Year: 2009; Article ID: 09-0738
DOI: 10.3201/eid1512.090738; TOC Head: Dispatch

Appendix Table. Epidemiologic and 23-locus VNTR data for 15 isolates of *Burkholderia mallei* from the Punjab province, Pakistan, and 9 *Burkholderia mallei* whole genome sequences*

Isolate	District	Type of equid	Collection year (mo)	Collection source	Clinical form	VNTR loci†																						
						20k	389k	397k	857k	933k	1500k	1690k	1764k	1788k	1934k	2050k	2065k	2170k	2341k	2356k	2445k	2666k	2815k	3091k	3145k	3152k	3564k	3652k
PRL1	F	Donkey	2002	Skin nodule aspirate	Pneumonic and cutaneous	9	7	13	10	6	Null	10	8	5	Null	17	12	18	3	6	3	2	6	10	5	23	23	6
PRL2	F	Horse (mare)	1999 (Jun)	Nasal swab	Pneumonic	9	9	13	10	10	Null	10	8	5	Null	20	13	24	3	6	3	2	6	10	5	24	23	13
PRL3	S	Horse (gelding)	2005 (Dec)	Skin nodule aspirate	Pneumonic and cutaneous	9	9	13	10	10	Null	10	8	5	Null	20	13	24	3	6	3	2	6	10	5	24	23	13
PRL4	S	Horse (gelding)	2005 (Dec)	Skin nodule aspirate	Cutaneous and nasal	9	9	13	10	10	Null	10	8	5	Null	20	13	24	3	6	3	2	6	10	5	24	23	13
PRL7§	F	Horse	2000	Skin nodule aspirate	Cutaneous and nasal	10	6	12	11	8	Null	10	8	6	Null	26	10	25	3	9	3	2	6	9	8	19	23	12
PRL11	F	Horse	1999 (Jun)	Skin nodule aspirate	Cutaneous and nasal	9	9	13	10	10	Null	10	8	5	Null	19	13	21	3	6	3	2	6	10	5	24	23	12
PRL13	F	Horse	1999 (Jun)	Skin nodule aspirate	Cutaneous and nasal	9	9	13	10	10	Null	10	8	5	Null	19	13	21	3	6	3	2	6	10	5	24	23	12
PRL19	L	Horse (gelding)	2005 (Sep)	Skin nodule aspirate	Cutaneous	9	9	13	10	10	Null	10	8	5	Null	Null	13	24	3	Null	3	2	6	10	5	24	Null	13
PRL20§	L	Horse (gelding)	2005 (Sep)	Blood	Septic; pneumonic, cutaneous, and nasal	9	7	13	10	6	Null	10	Null	5	Null	17	12	18	3	6	3	2	6	10	5	23	23	6
PRL33	F	Donkey	2007	Skin nodule aspirate	Pneumonic and cutaneous	9	9	13	10	10	Null	10	8	5	Null	19	13	21	3	6	3	2	6	10	5	24	23	12
PRL34	F	Donkey	2007	Nasal swab	Pneumonic	9	9	13	10	10	Null	10	8	5	Null	20	13	24	3	6	3	2	6	10	5	24	23	13
PRL41	F	Mule	2006	Skin nodule aspirate	Neurologic and pneumonic	9	7	13	10	6	Null	10	8	5	Null	20	12	19	3	6	3	2	6	10	5	23	23	6
PRL42	F	Mule	2007	Skin nodule aspirate	Cutaneous	10	6	12	11	8	Null	10	8	5	Null	18	9	26	3	8	3	2	6	9	8	14	23	11
PRL44	S	Mule	2007 (Feb)	Nasal swab	Nasal	9	9	13	9	10	Null	10	8	5	Null	22	13	21	3	6	3	2	6	10	5	23	23	13
PRL45	F	Mule	2007	Skin nodule aspirate	Cutaneous and nasal	10	6	13	11	8	Null	10	8	5	Null	18	9	26	3	8	3	2	6	9	8	14	23	11
200272 1280§	NA	Unknown	Unknown	Unknown	NA	11	7	13	10	14	4	7	15	5	19	17	7	22	3	7	Null	2	4	Null	9	19	16	15
ATCC 23344§	NA	NA (human)	1944	Knee fluid, skin pustules, blood	NA	30	6	9	13	9	4	9	8	5	20	20	13	Null	3	9	3¶	2	4	9	7	24	21	4
FMH§	NA	NA (human)	Unknown	Unknown	NA	30	6	9	13	9	4	9	8	5	20	20	13	Null	3	9	3	2	4	9	7	24	21	4

JHU§	NA	NA (human)	Unknown	Unknown	NA	30	6	9	13	9	4	9	Null	5	20	20	12	Null	3	9	3	2	4	9	7	24	21	4
GB8 Horse 4§	NA	Horse	Unknown	Unknown	NA	34	6	9	13	9	4	9	Null	5	20	20	13	Null	3	9	3	2	4	9	7	24	21	4
NCTC 10247§	NA	Unknown	1960	Unknown	NA	10	7	17	10	6	4	9	Null	5	17	16	13	23	3	8	3	2	4	10	9	23	23	12
NCTC 10229§	NA	Unknown	1961	Unknown	NA	12	12	17	10	11	4	9	16	6	18	20	9	14	3	8	3	2	4	9	10	29	23	14
SAVP1§	NA	Unknown	Unknown	Unknown	NA	10	Null	Null	Null	5	Null	9	8	5	Null	21	11	23	3	7	3	2	5	9	5	15	23	9
ATCC 10399§	NA	Horse	1942	Lung	NA	19	5	9	11	11	4	9	Null	5	15	27	6	32	3	12	3	2	3	9	8	8	24	8

*VNTR, variable number tandem repeats; PRL, postgraduate research laboratory; F, Faisalabad; S, Sargodha; L, Lahore; NA, not applicable.
†For the 23 loci, data are given as repeat copy number following the methods from (*7*). For whole genome sequences, repeat copy number was determined by in silico genotyping using the defined repeat motifs as described in (*7*). Null indicates PCR or in silico failure, likely because of mutations in the priming sites or incomplete sequencing of VNTR regions and does not necessarily indicate absence of a VNTR region.
§Information on whole genome sequence available from (*8*).
¶This locus has also been used in (*5*) against this isolate. Using the methods and defined repeat motif for this locus from (*7*) our raw data and in silico data from the whole genome sequence suggest 3 copies of this repeat, whereas (*5*) reported 5 copies.
